# Pre and Postoperative Sexual Dysfunction in Patients with Leriche Syndrome—A Prospective Pilot Study

**DOI:** 10.3390/ijerph19053091

**Published:** 2022-03-06

**Authors:** Michał Tkocz, Anna Brzęk, Mateusz Marcinek, Violetta Skrzypulec-Plinta, Damian Ziaja

**Affiliations:** 1 Department of Urology, FMS in Katowice, Medical University of Silesia, 40-055 Katowice, Poland; mtkocz40@interia.pl (M.T.); matmarcinek@gmail.com (M.M.); 2 Department of Urology and Uroonkology, St. Barbara Hospital Politraumatic Centre, 41-220 Sosnowiec, Poland; 3 Department of Physiotherapy, School of Health Sciences in Katowice, Medical University of Silesia, 40-055 Katowice, Poland; dziaja@sum.edu.pl; 4 Department of Women’s Health, School of Health Sciences in Katowice, Medical University of Silesia, 40-055 Katowice, Poland; skrzypulec-plinta@tlen.pl

**Keywords:** endovascular treatment, erectile dysfunction, IIEF-5, Leriche syndrome, surgical treatment

## Abstract

*Background*: Recovery of normal arterial inflow in the lower limbs after Leriche’s syndrome surgery does not always improve erection. This study assesses the effects of Leriche syndrome on erectile and ejaculatory dysfunction in patients awaiting surgical treatment and the impact of treatment used on sexual dysfunctions. *Methods*: 35 men with Leriche syndrome aged 61.3 years (SD = 7.74) were assessed for erectile dysfunction. The patients were classified into three groups: aortofemoral bypass (group 1); stenting of the iliac artery (group 2) and aortobifemoral bypass (group 3). The patients were qualified for surgery based on the TASC II guidelines. Follow-up was done 3 months after treatment. *Results*: The mean preoperative IIEF-5 score was 14. 69 (+/− 5.30), with better preoperative scores obtained by 54.3% of patients. A total of 51.4% and 48.6% of patients, respectively, reported normal erection enabling satisfactory penetration and normal ejaculation before treatment. After surgical treatment, satisfactory erection was reported by 60% of all surgically treated patients, whereas the presence of ejaculation was reported by only 14.2% of patients. *Conclusions:* The IIEF-5 score is a tool for careful assessment of vascular erectile dysfunctions, it allows for the evaluation of erectile dysfunctions in relation to atherosclerosis risk factors. The treatment strategy used allowed for slight improvement as evidenced to erection but decreasing normal ejaculation.

## 1. Introduction

Aortoiliac (aortobifemoral) occlusive disease (also known as Leriche syndrome) is a condition that takes many years to develop, gradually leading to symptoms resulting from impaired perfusion of tissues supplied by the final segment of the abdominal aorta and iliac arteries. These slowly progressing changes not only lead to lower limb ischaemia, but also cause vascular erectile disorders of varying severity [1]. The rapid development of vascular surgery and a marked improvement in treatment outcomes, as evidenced in reduced mortality rates, allowed for the common use of surgical management involving the aortic bifurcation in different age groups of patients with Leriche syndrome [2].

Erectile dysfunctions developing as a consequence (complication) of surgical treatment of the aortofemoral segment are a known and discussed interdisciplinary issue. Surgical/endovascular management impacts erectile and ejaculatory disorders to a varying degree. Surgical revascularization may cause insufficient arterial inflow to the lesser pelvis and despite restoring adequate blood supply to the lower limbs, it causes erectile disorders of varying severity. Furthermore, iatrogenic damage to the lower abdominal plexus causes retrograde ejaculation. Damage to the autonomic neural plexuses surrounding the abdominal aorta, aortic bifurcation and the iliac arteries is one of the main causes of erectile disorders, impaired ejaculation in particular. On the other hand, the long-term course of the disease causes endothelial damage in the small vessels of the penis. Reduced blood supply to the cavernous sinuses consequently leads to impaired penile microcirculation with progressive cavernous tissue remodeling and irreversible damage to the cavernous sinus endothelium, hence achieving satisfactory erection is not always possible despite restored arterial inflow [3,4].

Moreover, the extremely important psychological aspect related to the intimate sphere (erectile dysfunction), which is often overlooked or only superficially discussed by patients, should not be disregarded. The use of validated questionnaire tools allows for the diagnosis of erectile disorders and implementation of appropriate treatment approach in these patients. Standard diagnosis based on the guidelines of the National Institutes of Health and the European Association of Urology involves thorough collection of medical history with objectivisation of subjective data based on the International Index of Erectile Function (IIEF) and its modified version, which is a culturally, linguistically and psychometrically valid diagnostic tool [5,6,7].

Our aim was to assess the change in erectile dysfunction after revascularization in relation to the efficiency of the internal iliac arteries. We also focused on erectile dysfunction occurring before and after surgery for Leriche syndrome in terms of internal iliac artery obstruction, and what effect revascularization had on sexual dysfunction.

## 2. Materials and Methods

### 2.1. Study Population and Protocol

In the years 2012–2013 in the Department of General Surgery, Vascular and Angiology of Hospital SPSZK Nr 7 treated a total of over 4984 patients including over 3289 men. A total of 35 men aged 61.3 years (SD = 7. 74) diagnosed with Leriche syndrome, who had their sexual functions assessed before and after revascularization, were included in this prospective study. Patients diagnosed with hypertension were only included in the study if they were under constant cardiologist supervision and taking medications from a modern therapy that did not impair erectile function (excluding β-blockers and diuretics). Men with arteriographically confirmed aortofemoral occlusion with Fontaine grade II or III limb ischaemia were qualified for the study [8].

Patients with active cancer, chronic kidney disease, history of pelvic or penile surgeries, receiving nitric oxide inhibitors, as well as those with NYHA grade III–IV cardiovascular and respiratory failure and chronic obstructive pulmonary disease (COPD) were excluded from the study. Patients were not screened for psychiatric disorders. Only the history of their absence was taken into account. The Leriche syndrome is a complex multidisciplinary problem at the end of which is the patient with abdominal aortic outlet obstruction and all its consequences. Patients were not using any medication to improve erectile quality. Characteristics of patients in terms of comorbidities is shown in Table 1.

The patients were classified into the following three groups: group 1 (*n* = 16)—aortofemoral bypass at the age of 61.8 years (SD = 6.7), group 2 (*n* = 8)—stenting of the iliac artery at the age of 59.7 years (SD = 10.3), and group 3 (*n* = 11)—aortobifemoral bypass at the age of 61.7 years (SD = 7.7).

### 2.2. Measures

All research procedures related to erectile dysfunctions were carried out by the same physician.

All patients qualified for treatment underwent basic laboratory tests, ECG with cardiac assessment, plain chest radiography, and arteriography of the abdominal aorta from the level of renal arteries. All patients underwent arteriography using Axiom Artis DTA (Simens) with nonionic contrast agent (Ultravist). Staging was done based on the diagnostic findings by the same team of surgeons and the radiologist. After a thorough analysis of medical history and radiological findings, the patients were qualified for treatment in accordance with the TASC II guidelines [9].

Medical history of erectile dysfunctions using the abridged version of the IIEF score (IIEF-5) was collected. The questionnaire contained five questions with a maximum score of 5 points for each question for a maximum total score of 25. According to the authors of the questionnaire, a score of 21 or less suggests erectile dysfunction requiring consultation with a doctor.

The questionnaire was performed twice: at baseline (before surgical intervention) and 3 months after surgical treatment (final examination), as in accordance with the study protocol.

Surgical procedures were performed under general endotracheal anaesthesia. Endovascular procedures were conducted under local anaesthesia. A detailed description of surgical endovascular procedures is presented in many available publications in this field [10,11].

### 2.3. Ethical Statement

The study was approved by the Bioethical Committee of the Medical University of Silesia in Katowice under resolution no. KNW/0022/KB1/30/12 (3 April 2012). The study conformed to the Helsinki Declaration. All patients provided written informed consent prior to the study, including enrollment and data collection.

### 2.4. Statistical Analysis 

For missing data, the last observation carried forward (LOCF) method will be used. This analysis imputes the last value observed before dropout, regardless of when it has occurred. Quantitative data will be described using mean values and standard deviation. Homogeneity between samples will be examined using the Kolmogorov-Smirnov 2-sample test and if necessary, supplemented by the Lilliefors revision. It is planned to calculate 95% confidence intervals (CIs). The hypotheses were verified using the non-parametric Mann–Whitney U test and Kruskal–Wallis test, as well as Wilcoxon matched-pairs test and the McNemar’s test for dependent variables. The power calculation of the test with an assumed sample of 35 people was calculated at 0.92. Probability value (*p*-value) < 0.05 indicated that the result was statistically significant.

## 3. Results

The mean preoperative IIEF-5 score was 14.69 (±5.30), with better (by 2.29 ± 2.0) preoperative scores obtained by 54.3% of patients. 

A total of 51.4% and 48.6% of patients, respectively, reported normal erection enabling satisfactory penetration and normal ejaculation before treatment. After surgical treatment, satisfactory erection was reported by 60% of all surgically treated patients, whereas the presence of ejaculation was reported by only 14.2% of patients. Considering the same risk factors leading to Leriche syndrome and a history of erectile dysfunction, a relationship between intermittent claudication and the lack of erection, which was reported by 54.2% of patients before surgical treatment, was taken into account. The distribution of pre- and postoperative erection and ejaculation is shown in Table 2.

Preoperative radiology with particular focus on the patency of the iliac arteries, which was performed in all patients, provided extremely valuable information (Table 3).

Patency of both internal iliac arteries, one artery and obstruction of both arteries were found in 37.1%, 28.5% and 34.2% of patients, respectively. No statistically significant differences in erectile dysfunction measured with the IIEF score before (*p* = 0.49) and after surgery (*p* = 0.48) were found between the compared groups. The lowest means and standard deviations were reported for group 1 (Table 4).

**Figure 1:**
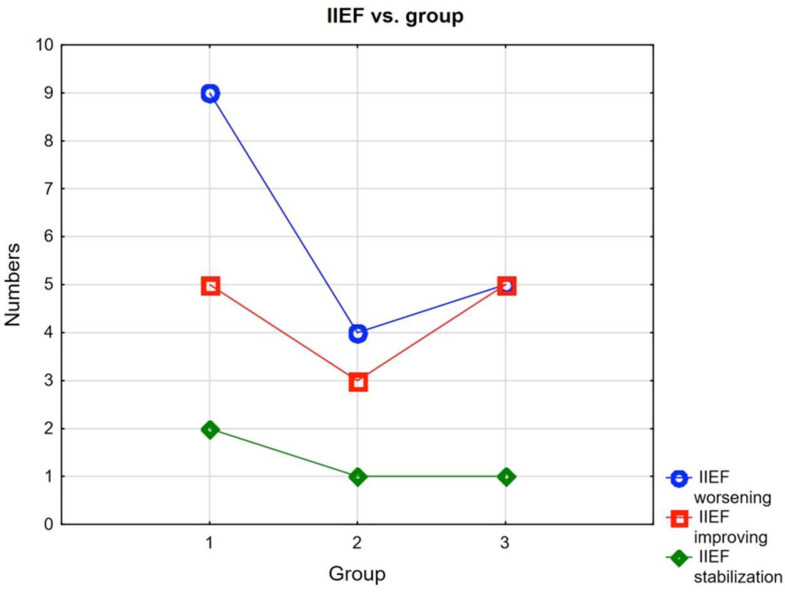
Interaction IIEF vs. groups.

In the study group, only 4 men did not require treatment for erectile dysfunction before the procedures, while only 2 did after. However, improvement in scores was obtained by 13 patients and stabilization of scores by 4 patients (Figure 1).

An analysis of the impact of hypertension on erectile dysfunctions showed an inverse relationship after surgical treatment (*p* = 0.05). A difference in pre- and postoperative scoring was found in hypertensive patients (*p* = 0.03); this difference was not spectacular in non-hypertensive patients (Table 5).

Among the study patients, only 5 had diagnosed and controlled type II diabetes Mellitus. All of them also had hypertension in terms of SBP (X = 128, SD = 8.37), DBP (X = 74, SD = 5.48). Two of them were diagnosed with erectile dysfunction before surgery. These were patients with Fontaine grade III. All had a history of cigarette smoking, although were currently not.

## 4. Discussion

### 4.1. Sexual Disfunction vs. Methods to Restore Blood Flow in the Lower Limbs

Therapeutic advances are inseparably associated with the development of novel minimally invasive diagnostic methods allowing for detailed visualization of aortofemoral lesions. Patients with Leriche syndrome expect not only restoration of adequate blood flow in the lower limbs, but also resolution of erectile dysfunctions that accompany this syndrome. The IIEF scale was used in this study to assess subjective complaints about erectile quality in patients with Leriche syndrome. We attempted to answer the question how improvement/restoration of abdominal aortic cross-section in these patients influences erectile quality. The average age of the patients studied (geriatric) suggested multi-morbidity and often the use of multiple medications. The possible relationship between ED and age was already suggested in 1948 by Kinsey [12], and was later confirmed by Feldman et al. [13] in their research, which was a continuation of the Massachusetts Male Aging Study (MMAS). Maintenance or restoration of adequate blood supply in the internal iliac arteries was the basis for attempts at internal iliac artery revascularization using extra-anatomic bypass grafts by De Palme, Marchant, Cronenwett, and Schuler [14,15,16,17]. The presence of preoperative ejaculation was reported in in nearly half of patients undergoing surgical treatment in a comparison that generally declined after surgery. Ejaculation was not restored in any of the patients who had no ejaculation before treatment, and further several patients lost ejaculation as a result of surgery. The authors asked themselves what caused this, pointing unequivocally to surgical treatment, which could cause damage to the autonomic nerve branches responsible for controlling normal ejaculation. In the 1970s, Sabri and Cotton suggested that damage to the branches of the lower abdominal plexus descending along aortic bifurcation and its division may lead to retrograde ejaculation after surgical treatment for Leriche syndrome. In the years that came, many authors agreed that careful isolation of the anterior abdominal aorta and its bifurcation allows for maintaining proper sperm emission [18,19]. A question should be asked whether changes in penile microcirculation reflect the degree of damage to the vascular tree (the internal iliac arteries, the internal pudendal arteries, the cavernous arteries, and the helicine arteries). Maintaining adequate arterial inflow with undisturbed neurogenic and venoocclusive mechanisms is associated with patent internal iliac arteries and abundant collateral circulation in the lesser pelvis.

### 4.2. IIEF-5 Scores

Preoperative assessment of erectile dysfunctions using the IIEF-5 score and considering the type of surgery showed no statistically significant differences between patients undergoing three different types of procedures. The authors were not troubled by the result of lack of statistical significance in terms of IIEF scores. Of far greater practical importance is the improvement of a few points or stabilization after the treatments, even though these patients still require treatment due to a serious course of multiple diseases. Minor improvements on individual questions have an impact on patient well-being. Critical assessment of the IIEF-5 scores may imply that the scale may be considered as an unsatisfactory diagnostic tool used for the assessment of organic (vascular–arterial) erectile dysfunctions. The study group included patients in whom despite adequate arterial blood supply to the lower limbs, the endothelial defect could be responsible for the lack of adequate erection, as self-assessed by the patients. There are many reports in the available literature on erectile dysfunctions assessed with the IIEF-5 score, which confirm the impact of cultural, religious, regional, and educational factors on the obtained score. Literature data indicate that IIEF-5 is a useful diagnostic tool allowing for the assessment of changes in patients with erectile dysfunction as a result of pharmacotherapy. Brandler et al., showed that its utility in the differentiation of different forms of disorders is limited compared to objective diagnostic methods [20]. The problem of lack of ejaculation in patients was noticeable both before and after the procedure. This is called retrograde ejaculation. It should be remembered that lack of ejaculation can also occur as anejaculation, but such patients were not recorded in our study.

### 4.3. Leriche Syndrome vs. Multi-Disease and Sexual Dysfunction

Erectile disorders in Leriche syndrome are very complex and associated with multiple factors contributing to atherosclerosis leading to occlusion of the distal abdominal aorta as well as comorbidities, such as hypertension, which may cause endothelial damage and, consequently, penile microcirculatory impairment, diabetes mellitus, as well as with ageing, which contributes to the severity of atherosclerosis of vessels supplying the penis. The patients in our study characterized the multi-disease groups discussed. In their experimental study in rats, Hannan et al. showed that ageing-related changes involve remodeling of the pudendal arteries, which impairs cavernous hemodynamics, causes damage to vascular endothelium and cavernous sinuses, as well as arterial remodeling with thickening of the internal arterial membrane and impaired arterial relaxation [21].

Erectile dysfunction and cardiovascular diseases share common risk factors and mechanisms leading to endothelial dysfunction. Precise knowledge and understanding of the mechanisms leading to endothelial dysfunction, including penile endothelial dysfunction, has led to a change in the view of erectile dysfunction pathophysiology and consequently to a change in treatment. The introduction of inhibitors of phosphodiesterase type 5 has changed the face of erectile dysfunction treatment and consequently improved patients’ quality of life. Patients with Leriche syndrome constitute a heterogeneous group in which the predominant symptom is variously expressed obstruction of the terminal segment of the abdominal aorta. Abnormal penile perfusion or impaired penile arterial blood flow in this syndrome is a consequence of damage to the penile microcirculation and, consequently, of all mechanisms leading to the development of Leriche syndrome. The duration of the lesions is also important, as they cause changes in large vessels such as the abdominal aorta or its bifurcation, affecting penile perfusion and damage to small vessels. It seems to be very important to search for an answer whether effective restoration of circulation in the terminal segment of the abdominal aorta and iliac vessels and thus restoration of an adequate inflow of arterial blood to the penis will improve the quality of erection and thus the quality of life of patients.

Confirmation that the basis of erectile dysfunction in Leriche syndrome lies in damage to the endothelium of the small penile vessels is the analysis of the results of a very extensive study by Viigamma et al., from 2020. The authors confirmed the influence of hypertension and its treatment of erectile dysfunction with particular emphasis on modern treatment with drugs of non-deteriorating profile. This paper hypothesizes the association of erectile dysfunction as one that may precede the onset of symptoms of coronary artery disease. The explanation for this process is related to the diameter of the arterial vessels and the possibility of the development of atherosclerotic lesions. Atherosclerotic lesions appear faster in small vessels, in this case in penile vessels [22].

In own study non-hypertensive patients scored, on average, higher. According to Klonler, 67–68% of hypertensive patients reported erectile dysfunctions of varying severity [23]. In the Massachusetts Male Aging Study (MMAS), Feldman et al., showed that 15% of patients treated for hypertension experienced erectile dysfunctions [13]. In 2000, Burchardt performed an analysis of IIEF scores in patients treated for arterial hypertension and confirmed that erectile disorders were more likely to develop and were more severe in these patients compared to healthy men [24]. Giuliano et al., showed that erectile disorders affect 67% of patients with confirmed arterial hypertension, whereas Sun et al., found erectile dysfunctions in 41% of hypertensive patients compared to 19% of men without concomitant erectile disorders [25,26]. Safetel and his team analyzed database of more than 200,000 patients with erectile dysfunctions and confirmed arterial hypertension in 42% of these patients [27]. Hypertension may cause damage to the endothelium of small penile vessels and irreversible erectile dysfunction in patients with Leriche syndrome [4].

Type II diabetes mellitus is also one of the important factors of erectile dysfunction in Leriche syndrome. In our study, diagnosed in only five cases, controlled by specialists. The authors point out the need for long-term follow-up of patients with type II diabetes mellitus. Active prophylaxis of cardiac complications is necessary for improvement of long-term survival of patients after vascular reconstructive surgery.

### 4.4. Limitation of the Study

Our study has some limitations, with a slightly under-representative group being the major one. However, it should be emphasized that the study was interventional and longitudinal in nature, hence multiple exclusions at each stage of research. The limitations also motivated us to undertake an in-depth multifaceted analysis of patients with Leriche syndrome, including the physical activity component, prevention, comorbidities, as well as the psychological sphere and social contacts.

## 5. Conclusions

The IIEF-5 score is a tool for careful evaluation of erectile dysfunctions, especially vascular ones. It allows for the evaluation of erectile dysfunctions in relation to the risk factors for atherosclerosis. Patency of the internal iliac arteries, and the presence of collateral circulation may enable adequate functional blood flow in the cavernous sinuses. The treatment strategy used allowed for slight improvement as evidenced to erection allowing for penetration on the one hand and decreasing ejaculation on the other.

The research has which implications for practice, which in the opinion of the authors, it should be included long before the onset of symptoms, perhaps as early as early adulthood and should also be extended to the partners of men.

## Figures and Tables

**Table 1:** Comorbidities in the study group.

Comorbidity	*N* (%)
HT (hypertension)	31 (88.6)
IHD (ischaemic heart disease)	17 (48.6)
IM (myocardial infarction)	8 (22.9)
PU (peptic ulcer)	5 (14.3)
SD (stroke disease)	1 (2.9)
DM (diabetes mellitus)	5 (14.3)

**Table 2:** Numerical distribution of pre- and postoperative erection and ejaculation.

*n* (%)	Erection	Ejaculation
Before yes	20 (57.2)	15 (42.7)
Before no	15 (42.7)	20 (57.2)
After yes	21 (60.0)	5 (14.3)
After no	14 (40.0)	30 (85.7)
Before yes/after yes	17 (48.6)	5 (14.3)
Before no/after no	13 (37.1)	18 (51.4)
Before yes/after no	1 (2.9)	12 (34.3)↓
Before no/after yes	4 (11.4)↑	0 (0.0)

**Table 3:** Pre- and postoperative erectile and ejaculatory disorders depending on the degree of internal iliac artery patency.

Comorbidity	Before Treatment		After Treatment	
	Erection	Ejaculation	Erection	Ejaculation
Both patent (*n* = 13)	9 (25.7%)	4 (11.4%)	10 (28.6)	1
One patent (*n* = 10)	6 (17.1%)	9 (25.7%)	9 (25.7%)	4
Both obstructed (*n* = 12)	3 (8.6%)	2 (5.7)	2 (5.7%)	0

**Table 4:** Pre- and postoperative IIEF scores in relation to the type of intervention.

Type of Procedure	Before Treatment		After Treatment	
	X (SD)	Min–Max (Me)	X (SD)	Min–Max (Me)
Group 1	13 (4.10)	9–21 (12.5)	13.06 (4.73)	5–21 (12)
Group 2	16.13 (5.36)	10–23 (15)	15.75 (9.90)	9–23 (15)
Group 3	15.36 (6.79)	7–24 (17)	13.83 (6.0)	5–21 (15)
*p* *	0.49		0.48	

Abbreviations: Group 1—aortofemoral bypass, Group 2—iliac stenting, Group 3—aortobifemoral bypass, * for Kruskal–Wallis test.

**Table 5:** Pre- and postoperative IIEF scores, in the group of patients with and without HT.

Type of Intervention	Before Treatment		After Treatment		*p* **
	X (SD)	Min–Max (Me)	X (SD)	Min–Max (Me)	
HT	14.44 (5.51)	7–24 (12.5)	14.26 (5.42)	5–23 (13)	0.03
Non HT	18.25 (5.51)	16–20 (18.5)	18.0 (2.16)	16–21 (17.5)	0.23
p *	0.18		0.15		

Abbreviations: HT—hypertension, nonHT—no hypertension, * for Mann–Whitney U test with continuity correction, ** for Kruskal–Wallis test.

## Data Availability

Data are archived at the Medical University of Silesia in Katowice. If necessary, contact the first author by e-mail mtkocz@interia.pl.
